# Fractality of sensations and the brain health: the theory linking neurodegenerative disorder with distortion of spatial and temporal scale-invariance and fractal complexity of the visible world

**DOI:** 10.3389/fnagi.2015.00135

**Published:** 2015-07-15

**Authors:** Marina V. Zueva

**Affiliations:** The Division of Clinical Physiology of Vision, Federal State Budgetary Institution “Moscow Helmholtz Research Institute of Eye Diseases" of the Ministry of Healthcare of the Russian FederationMoscow, Russia

**Keywords:** fractal therapy, dynamical chaos, fractality of sensations, reactivation of brain plasticity, treatment and rehabilitation, aging, neurodegenerative diseases, amblyopia

## Abstract

The theory that ties normal functioning and pathology of the brain and visual system with the spatial–temporal structure of the visual and other sensory stimuli is described for the first time in the present study. The deficit of fractal complexity of environmental influences can lead to the distortion of fractal complexity in the visual pathways of the brain and abnormalities of development or aging. The use of fractal light stimuli and fractal stimuli of other modalities can help to restore the functions of the brain, particularly in the elderly and in patients with neurodegenerative disorders or amblyopia. Non-linear dynamics of these physiological processes have a strong base of evidence, which is seen in the impaired fractal regulation of rhythmic activity in aged and diseased brains. From birth to old age, we live in a non-linear world, in which objects and processes with the properties of fractality and non-linearity surround us. Against this background, the evolution of man took place and all periods of life unfolded. Works of art created by man may also have fractal properties. The positive influence of music on cognitive functions is well-known. Insufficiency of sensory experience is believed to play a crucial role in the pathogenesis of amblyopia and age-dependent diseases. The brain is very plastic in its early development, and the plasticity decreases throughout life. However, several studies showed the possibility to reactivate the adult’s neuroplasticity in a variety of ways. We propose that a non-linear structure of sensory information on many spatial and temporal scales is crucial to the brain health and fractal regulation of physiological rhythms. Theoretical substantiation of the author’s theory is presented. Possible applications and the future research that can experimentally confirm or refute the theoretical concept are considered.

## The Theory

Comparison of the known facts, phenomena and relationships, and logical analysis makes it possible to assume that the spatial–temporal structure of our incoming sensory information represents the key driver of the healthy development of the brain. Man is a subject and object of the dynamic chaos of nature. Natural fractals accompany us throughout our lives. Additionally, all the wealth of sensations that we receive, enjoying the results of other people’s creativity and in the process of our creative thinking we can also refer to the “fractality of sensations.” Distortion of the fractality of sensations may be a factor contributing to the weakening of brain’s functions, distortion in cognitive performance and dynamics of gait by reducing the capacity of adaptive plasticity.

In this article, an objectively existing relationship between normal functioning or pathology of the brain and the spatial–temporal structure of the visual, auditory, and other stimuli that affect people throughout the life was proposed to exist and theoretically substantiated. The theory argues that the deficit or distortion of the fractal complexity of visual and other environmental influences may lead to anomalies of development and aging. In the same time, the use of fractal flickering and other sensory modalities of fractal stimulation helps to restore the function of the brain by acting via activation of the brain plasticity. The application of fractal therapy can provide particular benefits in the elderly, in patients with neurodegenerative disorders and amblyopia.

The following assumptions are derived directly from the theoretically established relationship:

• Fractal geometry and non-linear dynamics of complex processes are not just a characteristic property that is inherent for all natural (environmental and biological) systems. It is a sign that determines the quality of human life.
• Fractal geometry and a non-linear dynamics of physiological processes of the human body are not only typical signs of a healthy body. It is a key feature, whose preservation, maintenance, and recovery is essential to maintain physical and mental human health throughout the life, from birth to old age.

Below, we present data collected from different areas of expertise that are relevant to theoretical substantiation of the theory, including:

• Evidence of the characteristic non-linear structures in nature, of the fractal geometry and dynamics in a healthy human body and brain, and the distortion of fractality correlated to aging and pathology;
• The current understanding of the brain plasticity, dependent on the sensory experience, and neuroplasticity changes in diseases related to abnormal development and age-related conditions;
• The techniques used today to reactivate the plasticity of the adult’s brain, and recent new data that may indicate a possible usage of sensory stimuli with the non-linear temporal structure to influence the neuroplasticity.

We should note that the conclusion about the role of fractal therapy in the treatment of a variety of human diseases may be an outcome of an analysis of previously known scientific facts (i). Similarly, it can immediately follow the present theory of an objective relationship between the fractality of the environment (and our sensations) and brain health (ii).

However, the difference in logical reasoning is as follows:

(i) In the first case, the conclusion of the possible advantages of non-linear light therapy follows from the fact that the complex dynamics of some body functions usually follow the laws of deterministic chaos. In disease, this dynamics loses its complexity. Thus, it seems logical to try to recover the complexity of rhythms by using fractal entrainment cues to synchronize the dynamics of physiological fluctuations and external rhythms.
(ii) In the second case, the expected benefits of fractal stimuli in training and therapy follow not from typical non-linear dynamics of healthy physiological processes. Opposite, these benefits follow from an understanding of the need to preserve, maintain and restore a complex (fractal) dynamics. In certain situations, including diseases, this may need to use artificially created fractal environment.

## The Non-Linear World

### Characteristic Patterns of Fractal Geometry and Dynamics in Nature and Man

#### Natural Fractals and the Human Body

From the birth to deep old age, we live in a non-linear world, at which the scene objects and processes with the properties of fractality and non-linearity surround us. Against this background, the evolution of man took place. Against this background, all periods of our lives unfold.

The non-linear nature of physical objects is well-known today (Kirillov and Pelinovsky, 2013). Fractal geometry, which is capable to describe the natural objects, and non-linear dynamics also have been applied in the field of biology and medicine. Fractals are the irregular geometric figures or a set of points, possessing the features of self-similarity and a fractional dimensionality. Self-similarity and scale invariance are the basic properties of fractals. It means that the structure of the fractal object remains unchanged with the increase in the image regardless of the zoom (Mandelbrot, 1982; Feder, 1988; Weibel, 1991; Bassingthwaighte et al., 1994; Crownover, 1995; West et al., 1999). Nature example of the simplest mathematical fractal is a treelike structure with dichotomous self-similar branching, exhibiting properties of bifurcation. Natural fractals include the relief of the sea and river coastlines, of mountain ranges, the winding river networks. The Brownian crystal growth and lightning structure – all also belong to the natural fractals (Iannaccone and Khokha, 1996).

Natural fractal structures are the result of a process of self-organization in which the communication of structural levels of different scales occurs. Resulting natural fractal objects also have a self-similar structure, that is, with zooming such structures remain the same, regardless the scaling. Natural fractals belong to a class of statistical or random fractals. The fractal dimension sets the link between fractal structures and the properties of the environment. Fractal dimension usually exceeds the object topological dimension. Physical objects are rarely self-similar at an increase of more than four orders of magnitude (West et al., 1999). West et al. (1999, p. 1677) vividly noted: “Fractal-like networks effectively endow life with an additional fourth spatial dimension, the origin of quarter-power scaling that is so pervasive in biology.” We can often see new principles of self-organization in biological objects in case of an increase by two orders (Crownover, 1995).

Many complex structures of living systems exhibit fractal-like geometry. In a human body, a variety of complex anatomical structures has fractal geometry. They show the property of self-similarity at several scales (Goldberger et al., 1990, 2002; Weibel, 1991; Bassingthwaighte et al., 1994; Ayers, 1997; for review see Goldberger, 2006). Examples include the blood vessel branching, networks of the tracheobronchial tree and neural networks in the brain, the folds of the intestine, choroidal plexus, etc. Treelike fractals help describing and modeling of the tracheobronchial tree. Fractal structures are supposed to be very stable, because of their redundancy and irregularity (Goldberger et al., 2002).

#### Non-Linear Dynamics of Physiological Processes

For the description of dynamical, unpredictably time-varying systems, the concept of deterministic chaos is applied (Mandelbrot, 1982; Stewart, 1989; Crownover, 1995). The ‘chaos’ implies some definite properties of the deterministic dynamical system, the most important of which are a significant dependence of such system on the initial conditions, and its internal unpredictability. Any chaotic phenomenon we can describe with its trajectory in the analysis over time. The region in the phase space, in which the path of the system’s behavior is visually concentrated, is called chaotic (strange) attractor. The self-similar nature of the fractal processes can be qualitatively assessed with a graphical representation of their fluctuations at various time resolutions (Belair et al., 1995; Beuter et al., 2003). The concept of strange attractors has led to a new hypothesis of systemic properties of higher cognitive functions that depend on the dynamic interactions between parallel streams of information (Mattei, 2013).

Complex physiological processes also exhibit irregular fluctuations across multiple time scales and can be described using the theory of deterministic chaos. Fluctuations of physiological rhythms of a healthy body have the properties of fractals (Goldberger et al., 1990, 2002; Peng et al., 1993, 2002; Yamamoto and Hughson, 1994). Healthy physiological rhythms such as heart rate, respiration (Fadel et al., 2004) demonstrate complex variability that can be quantified using the fractal concept and the concept of non-linear dynamics. The self-similar nature of these fluctuations we can see when plotting at different temporal resolutions. Complex physiological dynamics allows the body to respond rapidly to internal and external perturbations of the environment (Lipsitz, 2004).

The special edition of Frontiers in Computational Neuroscience has included selected articles dedicated to the applications of non-linear and fractal analyses in different research fields in neuroscience and cognitive psychology (Mattei, 2014). The topic was assigned to ‘Non-linear and Fractal Analysis in Neuroscience and Cognitive Psychology.’ In this issue, the selected articles demonstrate usefulness of the non-linear analysis for modeling the healthy brain dynamics, for the diagnosis of neurological and psychiatric disorders, and monitoring of the therapeutic efficiency. The results of studies confirm that the advanced non-linear analysis helps to describe the complexity of brain dynamics accurately.

The healthy heartbeat interval was found to exhibit highly irregular behavior, fluctuating in a complex manner. Scaling analysis of cardiac interbeat time series demonstrated the presence of long-range fractal correlations in variations of the healthy heartbeat (Goldberger et al., 1990, 2002; Ivanov et al., 1999). The subtle, complex fluctuations of the inter-step (stride-to-stride) interval in the healthy human walking rhythm were also shown to exhibit the scale-invariance. Walking speed had no effects on the stride interval fluctuations and long-range correlations, indicating that the fractal variability in human gait in norm is quite robust (Hausdorff et al., 1995, 1996). While the dynamics of heartbeat has a chaotic pattern with signs of self-similarity, the tachycardia is relatively periodic and possesses a vivid rhythmic nature (cited from Goldberger et al., 2002).

In the spectrum of EEG of the healthy waking human brain the alpha rhythm dominates. Lehnertz (1999) presented an overview of studies that demonstrated a fractal dimension of alpha rhythm. Different works reported that the chaotic behavior is, likely, characteristic of the activity of the healthy brain areas, single neurons and neural networks (Babloyantz, 1989; Schiff et al., 1994; Das, 2001; Faure and Korn, 2001; Korn and Faure, 2003; Izhikevich, 2007).

The chaotic behavior is believed to make neurons able to switch quickly between different states (Schiff et al., 1994). It provides the flexibility of the central nervous system (CNS) and its resistance to external influences. The non-linear behavior of axons and excitable cells seems to have the regulatory role of inhibitory coupling once chaotic cells become members of larger neuronal assemblies (Rabinovich and Abarbanel, 1998). It has been also suggested that chaos may contribute to the neuronal code (Skarda and Freeman, 1987; van Vreeswijk and Sompolinsky, 1996; So et al., 1998).

#### Disruption in Fractal Dynamics of Physiological Processes

Various studies have found correlations between the aging process or disease and a loss of the complexity of many physiological processes. In pathological conditions and aging, ordered fluctuations in the parameters of physiological functions were reported (Lipsitz and Goldberger, 1992; Goldberger et al., 2002; Peng et al., 2002). Diseases tend to cause highly periodic dynamics of the process, which is dominant on the same time scale. Chaos in health allows the body to respond adequately to rapidly and unpredictably changing circumstances. Thus, the reduction of multiscale non-linear complexity of physiological functions in pathological conditions and human aging can potentially reduce opportunities for adaptation.

The disease is not always exhibited in an increase in the regularity of fluctuations. It might also lead to the extreme irregularity of changes, which, however, do not meet the criteria for non-linear chaos (Goldberger et al., 2002). Recent clinical study verifies that increasing age is associated with a reduction in the overall heart rate variability and complexity of physiological dynamics (Takahashi et al., 2012). These data were obtained using a Shannon entropy, conditional entropy, and symbolic analysis. In the aging process, the distributions of Shannon entropy patterns remained similar to younger subjects. At the same time, the patterns were more repetitive in the old group, indicating a marked change of autonomic regulation. A decrease in complex variability in the temporal patterns of heart rate that accompany aging and disease has been attributed to a breakdown of the underlying regulatory feedback mechanisms.

The fractal scaling showed disturbance in conditions associated with the disorder of breathing and obstructive sleep apnea (Goldberger, 1997; Goldberger et al., 2002). Recently, the band-limited transfer entropy analysis revealed a reduction in the high-frequency contribution of respiration to heart rate complexity with normal aging (Nemati et al., 2013). A fractal scaling in stride interval showed a reduction in the elderly. This was also reported for patients with pathological human walking (Dingwell and Cusumano, 2000) including PD (Hausdorff et al., 1997) and HD (Bilney et al., 2005).

Electromyography reflects the activity of the spinal motoneurons. As a non-linear signal, the surface electromyogram displays chaotic behavior and has a fractal dimension (Nieminen and Takala, 1996). The non-linear parameters of surface electromyogram signal in the PD patients significantly differ from the electromyogram in the healthy control persons (Meigal et al., 2009). Recently, Meigal et al. (2013) reported that the non-linear characteristics of surface electromyogram and tremor acceleration may have a possible diagnostic and predictive value for patients with PD.

The reduction in the multiscale complexity of the background brain activity has been shown in schizophrenia (Kotini and Anninos, 2002; Takahashi et al., 2010), epilepsy (Saermark et al., 1989), AD (Besthorn et al., 1995; Stam et al., 1996; Abásolo et al., 2005; Hornero et al., 2009), and PD (Stam et al., 1995; Anninos et al., 2000).

Three major effects of AD included slowing of the EEG, decrease in the EEG complexity, and disturbances in the EEG synchrony (Dauwels et al., 2010). Strong statistical evidence was obtained for a weak non-linearity in EEG in schizophrenia. Recent advances in methodology allow assuming that the “non-linear theory” of schizophrenia may be useful for the understanding of this disorder (Breakspear, 2006).

A branched vascular network of the normal human retina showed statistical self-similarity, exhibiting the properties of fractals (Family et al., 1989; Mainster, 1990; Daxer, 1993, 1995; Avakian et al., 2002; Masters, 2004). Several research groups presented strong evidence that the fractal dimension of blood vessels in the normal human retina is about 1.7. In many publications, the fractal analysis of the human retinal vasculature at different stages of DR showed mixed results (for review see Zueva, 2014). The contradictory results may occur because the study design was not always adequate to answer this question. The studies did not have the specific aim to determine the correlation between the fractal dimension of the retinal vasculature and the stage of retinopathy. For example, in a large cohort of diabetic patients with the early stages of DR, the fractal dimension of the retinal vasculature was analyzed in association with the presence or absence of signs of retinopathy (Cheung et al., 2009). However, these data usually were not compared with the fractal dimension of the retina in healthy individuals without diabetes. At the same time, the presence of diabetes, even in the absence of signs of DR in the fundus, can, possibly, lead to the alterations of anatomical structures, including the vascular network.

The reduction in the complexity of a dendritic branching and length in magnocellular and parvocellular layers of the lateral geniculate nucleus was recently discovered when modeling glaucoma in adult non-human primates (Ly et al., 2011). Blockade of *N*-methyl-D-aspartate receptors with memantine attenuated a decrease in the dendrite complexity and length in the relay lateral geniculate nucleus neurons in primate glaucoma (cited from Ly et al., 2011). Destruction of the dendritic branching is one of the characteristics and a potential mechanism of neurodegeneration not only for glaucoma. In AD, it may induce infringement of architecture of neural networks (Moolman et al., 2004). Disturbances to dendrite branching can disrupt the neural network organization and lead to the neural dysfunction, as in human neurological disorders including AD.

Hu et al. (2009, 2013) reported recently that in the AD patients with dementia, a parallel destruction of circadian rhythmicity and fractal patterns of activity is more pronounced in patients with greater quantity of amyloid plaques. These authors also showed in postmortem investigation that the degree of disruption in fractal activity is strongly associated with vasopressin and angiotensin-ergic neurons in the SCN. It suggests that the SCN affects the regulation of human action at multiple temporal scales and that the alterations in the fractal activity can be non-invasive biomarkers of neurodegeneration.

### The Fractality of Art

#### Fractal Architecture

Works of art created by man may also have fractal properties. Mandelbrot (1982) was the first who tried to distinguish the architectural styles in Euclidean and fractal geometry. The basic properties of fractal structures such as a self-similarity, fractional dimension, recursiveness, and discontinuity, were used in architecture by Peter Eisenman. Jencks, Kavannagh, Johnson, and Crowe also used them in their works (cited from Oswald, 2001). Oswald (2001) focused on 20 years experience in the implementation of fractal geometry in architecture and the main trends in the development and history of acceptance and rejection of the fractal concept. He presented an overview of the rise and fall of the fractal architecture in the late 20th century, noting prominent examples of historic buildings that exhibit fractal forms. Many historic buildings demonstrate an intuitive understanding of fractal geometry but do not constitute consciously created fractal architecture. They include medieval castles, Baroque churches, and Hindu temples. Oswald (2001) noted as an intuitive type fractal design also the works of Frank Lloyd Wright and Louis Sullivan. A project of Peter Eisenman ‘House 11a’ has been the first example of architectural art that uses the concept of fractal scaling.

Some examples of folk architecture, which were built by people at different times around the world, have fractal properties (Bovil, 1996; Batty, 2005). One can assume that the most beautiful cities in the world are fractals (Batty, 2005), including a plan of the city, streets, building facades and landscaping plan. The fractal approach would allow making housing that adapts naturally to the needs of residents in the growth and transport (Frankhauser, 2008; Harris, 2012; Parashar and Bandyopadhyay, 2014). Architect James Harris explicitly notes that high-rise housing and high-density architecture can lose their usefulness in the modern city and is limited to individual cells, as in a prison or in a computer that controls the world. He also noted that similar to ‘as the trees spread their leaves to catch the sun, cities have to unfold to give people the air and open space’ (Harris, 2012). Lu et al. (2012) reviewed recently the basic concepts of chaos theory and fractal geometry required for the architecture design, which can anticipate changes in the environment, providing adaptability and flexibility over time.

#### Fractal Painting

Art and musical compositions can also have a fractal dimension. An iterated function system is commonly used for generation of fractal art. However, the fractal pictures appeared long before the concept of deterministic chaos (Briggs, 1992; Barnett, 2009). These famous paintings include “The Great Wave off Kanagawa” by Katsushika Hokusai, abstract painting by Jackson Pollock “Blue Poles: Number II,” an abstract landscape by the Jenifer Bacon and Gottfried Mayer-Kress “Canyons and Mesas” (cited from Barnett, 2009; see also Taylor et al., 2002, 2006). Pollock dripped paint from a can onto vast canvases. Analysis of his patterns showed that they are fractals as “fingerprint of nature” (Taylor et al., 2002, 2006). Drip painting of Pollock has been attributed to the “Fractal expressionism.”

Just as is the case in architecture, paintings of many artists seem to be fractals, despite the fact that they could not know about it. When creating fractal art an artist draws fractals, but does not create them using his computer. Forsythe et al. (2011) considered the visual complexity for some time as a predictor of the significance of artistic works. This study detected the extent to which the perceived complexity of visual art can be successfully predicted using automated measurement of complexity. As the most successful predictor of visual complexity, Forsythe et al. (2011) noted GIF compression. Interestingly, the value of the fractal dimension had a larger dispersion in judgments about the perception of beauty in visual art than in the measurement of only the visual complexity, especially for abstract and natural images. Their findings also showed that the removal of color from the artistic image made observers unable to create a meaningful judgment about its beauty (Forsythe et al., 2011).

#### Fractal Music

As well as for the fractal painting, fractal music can be a created intuitively fractal (in fine art), or can be created artificially by various methods. Hsü and Hsü (1990, 1991) have found that the change of acoustic sound in the compositions by Bach’s and Mozart’s has the fractal geometry. More recently the fractal dimension of different kinds of music (rock, traditional, classical music, and others – 180 scores) Bigerelle and Iost (2000) analyzed in keeping with the time domain. They showed that the fractal dimension helps to distinguish between categories of music; i.e., music can be classified by their fractal dimensions according to its dynamic aspects. Earlier, there was revealed with the fractal motion method that the “best-sounding” music has a fractal dimension near 1.4 (Hazard et al., 1998–1999).

## The Plasticity of the Brain and Sensory Experience

### Brain Plasticity and Multimodal Integration

Neuronal plasticity occurring in the sensory and motor systems is the amazing ability of the brain that allows adapting to the constantly changing world during early development, and also in young and older adults. At all levels of the CNS, plasticity may be caused by the loss or excess of mono- and multimodal stimulation and injury. It can occur as the consequence of non-use or over-use, and learning new skills (cited from Moucha and Kilgard, 2006).

Neuroplasticity refers to changes in the neural circuits and synapses, including migration and integration of new neurons, neurite outgrowth, synaptogenesis, and the modulation of mature synapses. These occur in a variety of CNS levels due to changes in external and internal environment (He et al., 2006; Pascual-Leone et al., 2011; Wainwright and Galea, 2013). These changes can develop due to the injury to the brain with large-scale cortical remapping, and alterations in behavior, emotions, and thinking.

Neuroplasticity is known to play a significant role in the development, learning, memory, and in recovery from brain injury. Developmental plasticity includes changes in neural connections due to brain/environment interactions, and cellular changes induced by learning. The predominant mechanisms of plastic alterations in CNS, which occur during development, include synaptic and homeostatic plasticity, and learning. Most synapses are highly plastic, and they change their strength under the influence of its activity and other forces. Synaptic plasticity is believed to play a role in the mechanisms of learning and memory (Black, 1998; Foehring and Lorenzon, 1999). Long-term potentiation and depression (synapse-specific Hebbian forms of plasticity) are related to the processes that regulate overall levels of neuronal and network activity. The experimental research has found several mechanisms that apparently monitor the levels of activity, such as a spike-timing dependent plasticity, synaptic scaling and synaptic redistribution (Abbott and Nelson, 2000). Homeostatic plasticity modulates the neural circuit activity and changes in the synaptic strength (Wierenga et al., 2006; Butz et al., 2009). So, it regulates the destabilizing effects of developmental and learning processes.

The brain is very plastic in its early development, but previously it was assumed that plasticity decreases throughout life, and the structure of the brain is relatively unchanged over the critical period during early childhood. However, this concept has been subjected to large revisions after numerous subsequent studies that have found various manifestations of the adult brain plasticity (Dragoi et al., 2000; Chen et al., 2011). The human brain is constantly changing, being in a state of reshaping each moment of the life (Eysel, 2002, 2009; Gilbert and Li, 2012; Sur et al., 2013). Moreover, the results of recent studies suggest that the development of cortex structure is never completed but shows continuing changes, which are intelligence-dependent (Schnack et al., 2014).

Neuronal plastic changes, which never stop, are studied the visual cortex. Experimental studies in animals indicate that visual cortex exhibits considerable plasticity during development (Hebb, 1947; Hubel and Wiesel, 1970; Gordon and Stryker, 1996; Espinosa and Stryker, 2012; Nagakura et al., 2013). Plasticity is also was found in adulthood (Dragoi et al., 2001; Chen et al., 2011; Rosa et al., 2013). Plasticity in the early development and in the adult visual cortex was hypothesized to share certain universal principles although mature synapse plasticity requires additional neurotransmitter-dependent mechanisms that alter inhibition and subsequently the response gain (Sur et al., 2013).

Spontaneous network spike activity in the brain and retina plays a significant role in the initial establishment of synaptic connections during development. It may establish a basis for subsequent learning and further refinement of neural circuits and brain connectivity. For example, spontaneous network activity in the retina prior to birth has been found to cause the formation of retinogeniculate connections (Feller, 2009). During critical periods in development, changes in the structure and function of developing neuronal circuits can be experience-dependent or independent of the environmental experience but undergo the influence of endogenous or exogenous factors (Black, 1998). After the critical periods, many factors guiding brain development, such as growth factors, have been shown to be downregulated. But they can be upregulated again in adulthood in response to lesions for re-activation of neuroplasticity (cited from Eysel, 2009). The adaptive plastic changes in the adult’s brain are typically space-limited to the level of axonal terminals and synapses. Therefore, the formation of new functional connections can be done by strengthening or weakening of existing synapses in the network (cited from Eysel, 2009). The neuronal reorganization will take place if the environment is modified during early stages of development, for example, following visual deprivation through eyelid suturing, or dark-rearing (Bengoetxea et al., 2012).

The ability to simultaneously use signals from several senses at the same time (in their synergy) is a fundamental aspect of the brain functioning. Multisensory integration, which synthesizes all details of the currently accessible information, provides a complete picture of the outside world. Development of multisensory integration occurs by the principles of associative learning. The ability to integrate different sensory information evolves as neurons acquire experience with the co-active cross-modal inputs (Yu et al., 2010; Xu et al., 2012; Stein et al., 2014). As an example, in the cortex of the cat, the maturation of healthy multisensory integration has occurred over an extended period of postnatal life. An experimental study in cats reared from birth to adulthood in the dark demonstrated that sensory experience is necessary for the maturation of the cortex multisensor circuits (Carriere et al., 2007). In the absence of multimodal experience, neurons do not develop the ability to integrate their inputs although capable to respond to multiple sensory modalities. The bond of different sensory channels with each other is absent in neonates. The emergence and maturation of multisensory integration depend on the content of the early sensory experience, changing and optimizing neural networks in the brain for the adaptation of animals and humans.

There is an increasing experimental evidence that sensory deprivation is associated with cross-modal neoplastic changes in the brain. Upon visual or auditory deprivation, brain regions that typically are associated with these sensory modalities begin to involve the intact sensory modalities (Wallace et al., 2004; Wallace and Stein, 2007; Merabet and Pascual-Leone, 2010; Mereditha et al., 2012). In dark-reared cats, a recent study has revealed significant modifications in temporal dynamics of the receptive field structure and the integrity of superior colliculus multisensory neurons. These modifications included discharge duration, peak and the average rate of spiking, as well as significant changes in the frequency of spontaneous activation and the degree of multisensory integration (Royal et al., 2010). These authors performed an extracellular recording of electrical activity of neurons in the multisensory deep layers of the superior colliculus in kittens reared in complete darkness until adulthood and then returned to the usual environment for an equivalent period. The results emphasized the importance of early sensory experience in the establishment of normal architecture of multisensory processing and highlight the plastic potential of adult multisensory circuits.

Cross-modal experience in the early period of animal life is likely to define the integration of stimuli of different modalities. Periodical exposure of cats reared in darkness to visual and auditory stimuli appearing randomly in space and time was insufficient to encourage maturation of the ability for cross-modal integration. At the same time, the exposure to spatiotemporally concordant cross-modal stimuli was very efficient (Xu et al., 2012). In a recent investigation, animals were also reared in the constant omnidirectional noise. Xu et al. (2014) tested whether cross-modal co-activation is sufficient for visual-auditory superior colliculus neurons integration or a co-variation experience is needed. The data testify that experience with covarying stimuli is the critical factor for multisensory maturation, maybe not only in the superior colliculus, but throughout the brain. It also indicated that disturbances in one sensory modality can have an adverse impact on the ability of the brain to associate multisensory information, which was shown earlier for perturbation of visual sensitivity. In the patients deprived of early visual input by bilateral congenital cataracts the alteration in the development of multisensory functions after a period of visual deprivation has been shown (Putzar et al., 2010). In this study, the cataract patients exhibited impaired audio-visual interaction compared to normally sighted controls. It suggests that visual input is a prerequisite in early infancy for healthy development not only of visual, but also of multisensory functions.

Original and review reports (Rapp and Heindel, 1994; Grady, 2008; for review, see Mozolic et al., 2012) and a meta-analytic review (Rhodes, 2004) have shown that widespread changes in the anatomy of the white and gray matter, neurochemistry, and functional activity occur in the aging human brain. These changes correlated to significant changes in all sensory systems. Multisensory integration also alters with age. Surprisingly, neurons retain sensitivity to cross-modal experience late in life, far after the normal developmental period for acquiring multisensory integration capabilities (Yu et al., 2010).

Studies of various design have shown that the multisensory processing provides larger improvement of performance in older than in younger adults (Helfer, 1998; Sommers et al., 2005; Laurienti et al., 2006; Peiffer et al., 2007; Diederich et al., 2008; DeLoss et al., 2013). Using a two-choice audiovisual discrimination task, Laurienti et al. (2006) showed larger improvement of response time for multisensory target compared to the mono sensory purpose for the elderly than for younger adults. Diederich et al. (2008) analyzed saccadic reaction time in older and younger adults to the switch-on visual stimuli presented with and without an accessory auditory stimulus. The responses in elderly subjects were considerably slower than in the more youthful observers. However, the decrease in mean response time to bimodal stimuli compared to the single visual stimuli was more prominent in the elderly participants (Diederich et al., 2008).

Visual cortex plasticity results from a complex interplay between the individual’s genetic background and the environment (Maya-Vetencourt and Origlia, 2012). Childhood environment effects, favorable or unfavorable, such as childhood neglect, interfere with all the development processes of the CNS. These events include neurogenesis, the formation and branching of neuronal processes, synaptogenesis, refinement of synaptic circuits, and myelination. The development of synaptic pathways usually occurs by the rule “use it or lose it” (Perry, 2002; Shors et al., 2012). That is, not only genetic, but also environmental aspects (“nature and nurture”) influence these neural processes.

### Neuroplasticity in Aging and Disease

Brain aging is believed to be reversible because the brain can re-structure itself through learning experiences, being plastic at all stages of life (Mahncke et al., 2006). Adult education and participation in different training activities are considered to be essential for the extension of the mental health (Guglielman, 2012).

#### Amblyopia

The insufficient multi-sensory experience is believed to play a crucial role in the pathogenesis of amblyopia. This developmental disorder occurs during a period of neural plasticity and is often considered irreversible in adults (Holmes and Clarke, 2006; Barnes et al., 2010).

Bonaccorsi et al. (2014) reviewed recently current experimental studies dedicated to the recovery of neuronal plasticity in amblyopia. The results obtained in the rat model have hinted at a wide potential of visual perceptual learning for recovery of vision in adult amblyopic subjects.

Clinical research also found a significant degree of plasticity in the visual system of the adults with amblyopia (Levi and Polat, 1996; Polat et al., 2004; Levi, 2005). A prospective, randomized, masked, controlled study provided a high evidence that the perceptual learning lead to a twofold improvement in contrast sensitivity and visual acuity (Polat et al., 2004).

It suggests that perceptual learning reflects alterations in early neural processes localized beyond the site of convergence of the two eyes. Perceptual learning was assumed to operate via a reduction of internal neural noise and through more efficient use of the stimulus information (Levi and Li, 2009; Freiherr et al., 2013). A brief period of video-game play improved substantially various spatial vision functions of low-level and high-level visual processing, including visual acuity, spatial attention, and stereo acuity (Li et al., 2011).

Understanding the factors that predetermine the critical periods and principles of their opening and closure is expected to form the basis of new methods of therapy aimed at the improvement of visual deficits in children and adults with amblyopia. Such methods include ways for decreasing the levels of inhibition (Wong, 2012), constraint-induced therapy training (Taub, 2010; Taub et al., 2014), and many others.

#### The Aging Brain

Cognitive decline is accepted to be a common phenomenon during aging. There is also an alternative hypothesis that cognitive performance in old age may reflect the consequences of learning on information processing, being related to increased knowledge (Ramscar et al., 2014).

On the other hand, stereological principles of cell counting helped to reveal that changes that occurred during normal human aging were region-specific and more subtle than previously believed (reviewed by Burke and Barnes, 2006). These authors presented a systematic review of recent experimental and clinical studies on animals and human. The significant cell loss does not occur during normal aging. Plastic changes in dendritic complexity (dendritic branching and length) were even greater in aged individuals than in younger adults, and than in patients with senile dementia (Buell and Coleman, 1979; West, 1993). Burke and Barnes (2006) have reviewed functional alterations that occur during normal aging in the medial temporal lobe and the prefrontal cortex. Based on the data discussed, the authors suggest that the loss of neurons does not significantly contribute to age-related cognitive impairments. Alterations in synaptic connectivity and plasticity, Ca^2+^ homeostasis, gene expression, and network firing properties were found to contribute to the selective behavioral deficits observed in advanced age (Burke and Barnes, 2006).

Mahncke et al. (2006) define four main interrelated factors that determine the process of inevitable degradation of the brain. The authors note that reduced schedule of brain activity, noisy processing, weakened neuromodulatory control, and negative learning – all promote plastic changes in the brain and functional decline. With aging, significant changes occur in all sensory systems and the low-level and high-level cognitive functions are involved (Mozolic et al., 2012). Progressive losses in function across multiple systems accompany the changing of multisensory integration.

Several studies have reported recently that older participants showed greater multisensory integration relative younger subjects (Mahoney et al., 2011, 2014; Freiherr et al., 2013). It occurred despite the continuing attenuation of function of the individual sensory systems. One possible mechanism that can explain this phenomenon is the principle of inverse effectiveness. This event stated that as the responsiveness to distinct stimuli (unimodal performance) decreases, the strength of multisensory integration increases (Stein and Stanford, 2008; Holmes, 2009). Mahoney et al. (2011) first proved the facilitative effect of pairing somatosensory with visual stimuli in older adults. In their investigation, younger and older adult observers responded to randomly present unimodal stimuli (auditory, visual, somatosensory) and paired multimodal stimuli (auditory-somatosensory, auditory-visual and visual-somatosensory). The reaction time was significantly smaller to multisensory than to unisensory stimuli in both groups. Nevertheless, older adults showed greater shortening of the reaction time when processing visual-somatosensory information while younger observers demonstrated a significant increase in multisensory integration for auditory-visual and auditory-somatosensory stimulation.

#### Age-Related Diseases

Wilbrecht et al. (2010) have shown in electrophysiological experiments in rodents that the ability of neurons to adapt in response to the impact of internal and external signals depends on their plasticity. The electrophysiological and biochemical studies performed in experimental models have found that dopamine in the basal ganglia plays a crucial role in regulating long-lasting changes in synaptic strength (cited from Calabresi et al., 2009).

In humans, different aspects of neural plasticity have been independently associated with or contribute to the disease state (Wainwright and Galea, 2013). It was assumed that disturbance of neuroplasticity played a central role in such neurological disorders as PD and AD (Lewis and González-Burgos, 2008; Hu et al., 2009; Varea et al., 2012). Understanding the mechanisms of the plasticity of the adult’s brain related to multisensory experience and brain injury or degeneration may have high clinical and social relevance including the development of new ways for the reactivation of neuroplasticity.

The theory, which links cognitive changes typical of normal physiological aging to a functional distortion in the dopamine system projection to prefrontal cortex, is known (Braver and Barch, 2002). According to this theory, alterations in dopamine system function and then abnormal prefrontal cortex activation do impact on cognitive control, including working memory, attention, and inhibition. Dopamine plays a role in modulation of cell excitability and synaptic plasticity. Therefore, dopamine-dependent corticostriatal plasticity was suggested to underly the long-duration motor response to dopamine replacing therapy in the patients with PD (Zhuang et al., 2013). Patients with PD have disturbances in the perception and estimation of time (Parker et al., 2013). Dopamine deficiency in PD triggers the degenerative process associated with adaptive changes in neuronal networks. These changes include a compensatory overactivity of remaining dopamine neurons and functional or structural remodeling in other neuronal systems (Zhuang et al., 2013). The long-term dopamine-replacing therapy can trigger adaptive phenomena or, on the contrary, the appearance of side effects. Therefore, learning the mechanisms of neuroplasticity in PD and response to the therapy is necessary for the elaboration of more effective targeted therapies.

Several studies described functional plasticity of retinal ganglion cells in glaucoma, optic neuropathy of different genesis, and other retinal pathologies (Weber and Harman, 2008; Porciatti and Ventura, 2012).

Pattern electroretinogram reflects the electrical activity of retinal ganglion cells. Clinical and experimental findings suggest that PERG may be altered long before the reduction in the retinal nerve fiber layer (Porciatti and Ventura, 2012). Porciatti and Ventura (2012) used the concept of neural plasticity to simulate the reversible/inducible changes in the PERG during the critical period (stage of retinal ganglion cell dysfunction), which precedes their death. Zhou et al. (2014) suggested that retinal glial cell activation induced by acute high intraocular pressure may cause the process of retinal synaptic plasticity through affecting the expression of synaptophysin and other synaptic proteins. In the rat model of acute ocular hypertension, the increase in expression of synaptophysin across the retina was observed from the inner to the outer plexiform layer. Therefore, glial cells can be a new target to modulate retinal synaptic plasticity after retinal injury.

## Non-Drug Ways to Reactivate the Plasticity of the Adult Brain and New Prospects for the Use of Sensory Stimuli with Non-Linear Temporal Structure

### An ‘Active Life’ and Environmental Enrichment

Numerous works have shown the ability to reactivate adult’s neuroplasticity in a variety of ways (Maya-Vetencourt et al., 2008, 2011; Spolidoro et al., 2011; Prakash et al., 2014). Various studies have been developed to understand how physical activity and exercise influence the brain functioning, to identify mechanisms by which exercise can protect them, maintain and restore. Particular attention was paid to the training impact on the structure and function of the hippocampus (Shors et al., 2001, 2012). Neurons are continually born and added to the dentate gyrus throughout life (Altman and Das, 1965). However, aging causes changes in the hippocampal neurogenesis that may lead to cognitive decline with age (van Praag et al., 2002). The brain injury early in the development was shown to contribute to the emergence of health problems that manifest itself only in old age (Barnes, 1994; Grossman, 2014).

Physical activity seems to enhance the synaptic plasticity, increase vascular network complexity, and levels of neurotrophins (Shors et al., 2001). The known paradigm of training, based on the plasticity of the aging brain was designed by Mahncke et al. (2006), Merzenich (2013). The authors postulated that in order to reactivate the brain plasticity, the older people have to be involved in the intensive complex activity. Intense activity apparently strengthens neuromodulatory systems, increases reliability and power of cortical representations, and learning management in adults. Using of training programs based on brain plasticity showed that older adults could quickly learn and significantly improve memory (Mahncke et al., 2006; Smith et al., 2009; Berry et al., 2010). The training programs also enhanced the task performance in people with schizophrenia and other mental disorders (Fisher et al., 2009; Merzenich, 2013). Chapman et al. (2013) proved that cognitive training – the complex mental activity – also induces the neuroplasticity in healthy elderly subjects.

Mora et al. (2007) suggest that physiological aging occurs asynchronously in different areas of the brain. They also hypothesized that the impact of aging on the neurons, dendrites, synapses, molecular and functional plasticity can be modulated by environmental factors even in adults (Mora et al., 2007). EE is considered to be one of the promising methods for the reactivation of the adult brain plasticity. In the EE experimental paradigm, the animals are placed in an enrichment environment. The EE allows obtaining a much greater stimulation of cognitive, motor and sensory activity than standard laboratory conditions. Recently, Alwis and Rajan (2014) critically analyzed this experimental paradigm. In an early development and adulthood, EE profoundly affects the animal brain at the functional, anatomical and molecular level (Kempermann et al., 2002; Nithianantharajah and Hannan, 2006, 2009; Butz et al., 2009; Baroncelli et al., 2010, 2012; Sale et al., 2012). Short-term exposure to the EE leads to a significant enhancement of hippocampal neurogenesis associated with a substantial improvement in cognitive performance (van Praag et al., 2000, 2005; Kempermann et al., 2002). It makes it possible to suggest that the active interaction with the outside world may mediate its positive effects on the brain function through the impact on neuroplasticity. Kempermann et al. (2002) have reported that hippocampal neurogenesis in adult mice that lived in EE for 10 months since the age of 10 months was fivefold higher than in the control mice. It was accompanied by an improvement in locomotor activity and learning efficiency, exploratory behavior. However, the authors noted that the concept of EE in studies with inbred rodents cannot be easily applied to the human conditions.

In rats and mouse models, amblyopia can be induced by monocular deprivation during early development. It has been shown that adult amblyopic rats transferred to an EE setting for 3 weeks undergo a full recovery of visual functions (Sale et al., 2007, 2012). Restoration of plasticity in enriched animals accompanied a threefold reduction in GABA release, and the beneficial effects of EE were eliminated entirely by intracortical infusion of benzodiazepine diazepam. These findings emphasize the crucial role of GABAergic transmission reduction in the manifestation of these effects of EE. Insulin-like growth factor 1 involved in neurogenesis, neuronal differentiation, and synaptogenesis, can reactivate the experience-dependent plasticity of the visual cortex in adults by reduction of local GABA levels (Maya-Vetencourt et al., 2012).

The effectiveness of physical exercise and increase of social interactions and visual stimulations in the restoration of visual function has been studied recently in adult rats with amblyopia (Baroncelli et al., 2012). The equally good result, which consisted in restoration of the ocular dominance and visual acuity, was obtained for the intensive motor activity in a running wheel. The exposure of rats to an optical EE using a rotating fluorescent lamp for maximal stimulation of the V1 neurons also showed good results. The possibility to reactivate the adult visual cortex plasticity have been demonstrated in humans with amblyopia by usage of such EE approaches, as playing video games and visual perceptual learning (Levi and Li, 2009; Astle et al., 2011; Li et al., 2011; Green and Bavelier, 2012). Mainardi et al. (2013) summarizes the current ideas about the mechanisms of the environment-induced plasticity in the arcuate nucleus of the hypothalamus.

The correct combination of appropriate pharmacotherapy with the EE strategies was suggested to be a promising therapy for certain neurological disorders to improve an internal repair capacity of the brain (review by Foster et al., 2011; Sale et al., 2012). Foster et al. (2011) focused their recent study on the different lifestyle factors and possible pharmacological therapy aimed to reduce the risk for and to improve cognitive functions in mild cognitive impairment, AD, and other age-related disorders. Special attention was paid to the fitness training, which has the largest positive impact for executive (frontal lobe) functions (Foster et al., 2011).

### Noise Therapy and Stochastic Resonance

The phenomenon of SR is believed to underlie the therapeutic effects of noise for the number of pathological conditions mentioned below. It refers to the general phenomenon observed in non-linear systems when the intermediate level of activity improves detection of subthreshold signals by maximizing the signal-to-noise ratio. This phenomenon is fundamental for the physical and biological processes (Wiesenfeld and Moss, 1995; Russell et al., 1999; Pakdaman et al., 2001; Moss et al., 2004). SR occurs in any system where detection requires passing a threshold. It is described as the consequence of interactions between non-linearity, stochastic fluctuations and a periodic force (Chialvo et al., 1997; cited from Korn and Faure, 2003). Experimental studies have documented that SR can control the firing rates in crayfish mechanoreceptors, frog cochlear hair cells, and other sensory systems of animals (cited from Korn and Faure, 2003). SR was also shown to occur at the level of ion channels (Bezrukov and Vodyanoy, 1995). Different other aspects of SR role in the nervous system have been discussed (Korn and Faure, 2003; Tokuda et al., 2010).

Continuous variations in the membrane potential of neurons in the CNS are known as “synaptic noise.” It occurs due to the summation of intermittent inputs from presynaptic cells and the unreliability of synaptic transmission (Brock et al., 1952). Synaptic noise has been first assumed to be stochastic (Calvin and Stevens, 1967; Shadlen and Newsome, 1998). It was later assigned to a deterministic phenomenon that reflects the chaotic behavior of afferent inputs (see Korn and Faure, 2003 for review). During the aging, there is an increase of endogenous neuronal noise. Its interactions with external input noise were investigated with a stochastic gain-tuning model (Faure and Korn, 2002; Li et al., 2006). With aging, the cognitive system has a larger internal neuronal noise and less plasticity. Li et al. (2006) have shown that if we stimulate the aging system, it continues to demonstrate the overall effect of SR, which requires more external noise.

We usually consider noise detrimental to cognitive performance, but the increase of knowledge about the phenomenon of SR gives reason to study the possible useful properties of the external noise in different situations. Addition of noise in non-linear systems can amplify the detection of a subthreshold signal. Therefore, the influence of the noisy environment on cognitive performance was studied with a neurocomputational model of attention deficit hyperactivity disorder (Söderlund et al., 2007). Participants were asked to perform a task to verify a mini-performance memory, verbal task to check the memory productivity, both in the presence and in the absence of auditory white noise. Authors found the positive impact of noise on cognitive function in the group with attention deficit hyperactivity disorder. This effect was explained by the phenomenon of SR, whereas the noise distorted performance in the control group (Söderlund et al., 2007). Authors proposed that the noise in the environment introduces an internal noise in the neural networks through the perception system and facilitates performance by inducing SR in the neurotransmitter systems. An important role of SR has been demonstrated earlier for dopamine signaling in the brain (Li et al., 2006).

Söderlund et al. (2007) suggested that an optimal (for different circumstances) amount of noise may be beneficial for cognitive performance, in particular in hypodopaminergic states. They suggested existence of a link between the effects of noise, dopamine regulation, and cognitive performance (Söderlund et al., 2007). Authors assumed that the noise inducing the effect of SR must be continuous and have high energy levels at all frequencies, as it takes place with white or pink noise. The computational model of the concept of SR predicted the positive impact of background noise on the attention and performance (Sikström and Söderlund, 2007). Individual differences in dopamine were supposed to handle individual differences in the noisiness effects (Sikström and Söderlund, 2007). Exposure to auditory background noise has been recently shown to improve cognitive performance in inattentive school children while it distorted the performance of attentive children (Söderlund et al., 2010). These findings suggest that there is the ability to control cognitive performance by using the background white noise stimulation (at least in children with problems of attention).

The positive effects of background noise were also found in the elderly (Priplata et al., 2003), in patients with PD (Yamamoto et al., 2005), and other neurodegenerative disorders (Priplata et al., 2003; Pan et al., 2008; Söderlund et al., 2010). These effects are consistent with the theory that links the cognitive changes characteristic of normal physiological aging with decreased function of the dopamine system projecting in the prefrontal cortex (Braver and Barch, 2002). It has been found that SR modulates neural synchronization within and between functionally relevant brain areas (Ward et al., 2010), which may be a general mechanism of the brain functioning.

The therapeutic approaches based on the SR have been used in the treatment of gait disturbance in the patients with neurodegenerative disorders. Randomly vibrating insoles were designed to ensure coordination and gait in the elderly, patients with diabetic neuropathy, and in the period of rehabilitation after a stroke (Priplata et al., 2003; Costa et al., 2007; Ross, 2007). This treatment improved the equilibrium sense and the gait control in patients subjectively, and it caused objectively a significant increase in the fractal dimension and the complexity of the step-to-step interval fluctuations. Old participants showed greater improvement than young people. It was shown that 24-h noisy galvanic vestibular stimulation may be useful in the amelioration of akinesia symptoms in patients with central neurodegenerative disorders (Pan et al., 2008). It implies the possible mediation of the beneficial effect through the known effects of the vestibular nerve on the basal ganglia and limbic system. The 24-h noisy galvanic vestibular stimulation was apparently effective in improving the long-term heart rate dynamics in patients with multisystem atrophy and the dynamics of daytime activity in patients with PD (Yamamoto et al., 2005).

### Benefits of Music

Research and experience tell us that art can heal, change human physiology and perceptions of the world. Human behavior and physiology change from the state of stress in a situation of deep relaxation, from anxiety to inspiration, so we can assume that creativity alters brain function and our lives. Staricoff (2004) reviewed studies that examined how art affects human health. Her scientific report showed the positive role of different types of art in mitigating some pathological conditions and in the training of practitioners in the field of health.

Music is known to have different psychological and physiological impacts on humans, affects brain activity and EEG (Petsche, 1996; Yuan et al., 2000; Jausovec et al., 2006). Sink et al. (2011) by using fractal analysis and data mining techniques, found strong associations between a complexity of auditory signals in the form of synthetic music and the resulting multi-channel EEG responses. They noted that psychologists believe that there is a particular fractal dimensionality in nature. “When the incoming stimuli imitate this fractal dimension, the nervous system would resonate with this fractal dimension and show a particular pattern” (Sink et al., 2011). These authors suggested a significant mathematical association between auditory stimuli in the environment and physiological processes in the human body. Confirmation of this assumption would also be another valuable proof of our theory considering the importance of the multimodal external environment to maintain human health. Improving the perception of speech-in-noise is accompanied by neural changes in the auditory processing, which indicates the plasticity of the brain (Anderson, 2013). It allows the use of auditory training for individuals who have difficulties in perceiving of useful auditory information on the background noise. There were observed effects of music on the EEG power spectrum, closely related to man’s emotions (Yuan et al., 2000). Changes in the frequency components of the EEG power spectrum from the delta to the beta-2 band were analyzed in 16 regions in silence, during noise or when listening to music. A significant decrease was seen in the power of total alpha-1 while the power of total theta increased when listening to music.

Several studies have reported an increased spatial–temporal reasoning performance after listening to Mozart for 10 min, but not in all studies this effect was observed. Also, a positive impact was unstable and depended on spatial tasks (Jenkins, 2001). Jausovec et al. (2006) investigated the influence, which Mozart’s sonata for two pianos in D major (K. 448) has on brain activity in the process of solving spatial rotation tasks. The data were compared with those in participants who before and after the training listened to Brahms’ Hungarian Dance number 5. Those who listened to Mozart showed a better task performance than did the respondents of the relax group and Brahms’ music groups. They also displayed less complex EEG patterns and lower alpha-1 and gamma-band synchronization. Therefore, Mozart’s music, by activating task-relevant brain areas, can enhance learning of the spatiotemporal rotation tasks. The results support the priming explanation of the “Mozart effect.”

Earlier, Gordon Shaw suggested that if the activity of the mind may sound like music, we can use music to stimulate the brain. The music may have effect by activating the firing patterns similar to patterns of sounding music (cited from Campbell, 1997; see also Rauscher et al., 1995). Rauscher et al. (1995) hypothesized that the effect of music on intelligence can be explained by the fact that the hearing of complex music excites cortical firing patterns, which are similar to those used in spatial reasoning. Other hypotheses were also proposed for an explanation of this effect of Mozart music (Roth and Smith, 2008). For over two decades, numerous studies of “Mozart Effect” have been carried out, which confirmed or refuted increased intelligence while listening to music by Mozart. These studies reported a temporary increase in cognitive skills, or could not find a statistically significant “Mozart effect” (Allen and Blascovich, 1994; Roth and Smith, 2008). So, the high-quality evidence that would satisfy the requirements according to GRADE guidelines is not yet available (Balshem et al., 2011).

Contradictory results may be a consequence of the insufficiently adequate choice of control groups and study design. As a possible explanation, we can assume that the short-term effects of music on performance and physiology of the brain may occur because virtually all researchers used passive listening to Mozart’s music. This flaw in study design might predetermine the inconsistency in published studies. Perhaps in such circumstances the effect will vary in professional musicians and non-musicians, and people that differently perceive the music. All these factors will require experimental verification of the proper design. The impact of musical training on the solution of perceptual and motor tasks is associated with structural and functional changes that occur mostly in the brains of musicians compared to non-musicians (Bhattacharya and Petsche, 2005).

The results of various studies also encourage us to pay attention to the fact that the beneficial effect of music on the perceptual learning and higher cognitive functions depends on the severity of their initial distortion. Namely, the effects in older adults are better than in younger adults, and than in patients with neurodegenerative disorders performance improvement can be seen more frequently than in a healthy aging. Particular interest has recently attracted the impact of music on creative thinking and the role of the emotional state in our perception of the world.

The EEG data (Bhattacharya and Petsche, 2005; Fink et al., 2009; Fink and Benedek, 2014) and functional magnetic resonance imaging data (Kowatari et al., 2009; Berkowitz and Ansari, 2010; Ellamil et al., 2012) were used to characterize the creative thinking. These studies estimated the effects of musical improvisation, fine arts or other creative activity on the brain activity and functional connectivity in scientists and dancers. Creative ideation attracted an unusual pattern of neural processes that was not typical for traditional solutions (not originative) tasks (Kowatari et al., 2009; Berkowitz and Ansari, 2010; Ellamil et al., 2012). A relationship was found between the EEG alpha activity and creative thinking (Fink et al., 2009; Fink and Benedek, 2014). EEG alpha power varied depending on the creativity associated with the test requirements, and changed with the level of the individual capacity for creative thinking. The artists showed a much stronger synchronization in the short and long-delta range during the task of mentally creation of pictures, while non-artists showed improvement in the near-beta and gamma range (Bhattacharya and Petsche, 2005).

Interestingly visual perception of emotional stimuli was shown to be dependent not only on the prior knowledge, but also on the emotional state of the observer (Jolij and Meurs, 2011). Music can change the relationship between mood and visual perception. Apparently by manipulating top-down modulation of visual processing, music can ultimately alter the way we perceive the world.

The musical experience is believed to have a positive influence on the perception of speech in noise in young adults (Parbery-Clark et al., 2009; Kraus and Chandrasekaran, 2010). It was shown that compared with performance in young adults, the older musicians demonstrate an enhanced speech-in-noise perception (Parbery-Clark et al., 2011). This enhancement of speech understanding in the elderly is likely associated with a greater auditory-specific cognitive and perceptual performance. Previously, it has been also found that musical training reduces the age-related decline in hearing ability due to the enhancement of the central auditory processing (cited from Zendel and Alain, 2012). Musical experience downplays the reduction in neural precision (age-related delays in neural timing) that occurs during the natural aging process (Parbery-Clark et al., 2012).

Alain et al. (2014) analyzed recently the results of numerous investigations that describe the musical training benefits in the auditory cognition in young and elderly, including the creation of a mental representation of the auditory environment. The author paid attention to the results showing that musical training has a positive impact on neural mechanisms in young adults and exhibits long-lasting improvements in hearing and cognitive control. From these results, the assumption was made that musical training might counteract age-related changes in auditory cognition and delay the hearing decline that is commonly observed in aging.

The individualized piano instruction was reported to be an effective tool for prevention of age-related cognitive decline (Bugos et al., 2007). Many other studies testify that music training can be an effective strategy for rehabilitation of older people (Kraus and Chandrasekaran, 2010; for review see Kraus and White-Schwoch, 2014). However, the exposure to vocal or instrumental background (pleasant and arousing) music did not influence the verbal learning. It led the authors to suggest that participants ignored this background stimulation to focus on the verbal learning task (Jäncke et al., 2014). The background music was also used to distract the listener from the performance of the primary test (Salamé and Baddeley, 1989; Klatte et al., 1995; Parbery-Clark et al., 2009; Kraus and Chandrasekaran, 2010). But as we have noted above, the background white noise can improve cognitive function in inattentive subjects, but reduces the performance of attentive persons (Söderlund et al., 2010). Thus, we cannot exclude that the sign and strength of the impact of background music on cognitive function may also depend on individual characteristics, in particular, on the power of the internal neural noise. It seems important to check the probability an impact of these factors on the study result in the future research.

The healing effect of music on the auditory, visual and motor processing, cognitive and emotional state seem to be more prominent in different pathologies of the CNS than in normally aging individuals.

The observations suggest that music is likely to become a valuable tool in neurological rehabilitation. As an example, in post-stroke patients, even passive listening to music was shown to have the beneficial effect on memory and mood. Särkämö et al. (2008) demonstrated that everyday music listening during the early post-stroke stage can facilitate the recovery of cognitive functions and prevent the negative mood. Recently, Särkämö et al. (2014) found that after an acute middle cerebral artery stroke, regular listening to music can enhance cognitive recovery and improve mood. It also induces fine-grained neuroanatomical changes in the recovering brain. Authors performed a voxel-based morphometry analysis in patients with the acute stroke and after the 6-months period of rehabilitation. The patients listened to their favorite music or audio books or did not receive any material for an audition (Särkämö et al., 2014). Frontal and limbic areas in patients with the left hemisphere damage showed an increase of the gray matter volume greater in the musical group than in the verbal and in the control group. The remodeling of gray matter in the frontal areas correlated with enhanced recovery of cognitive functions. Both perceptual and motor timing showed improvement in patients with PD when using music cued gait-training (Benoit et al., 2014). This observation supports the idea that coupling gait to the rhythmic auditory cues in PD patients is based on a neural network involved in both the perceptual and motor timing.

Learning to play a musical instrument is very challenging, due to the involving of multimodal integration and higher order cognitive functions. In particular, active playing on a musical instrument can engage the sensorimotor system as well as the auditory system. Musical training involves simultaneously the motor system and a multisensory (auditory, visual, and somatosensory) perception. Very likely, it can serve as a useful model to study the multimodal brain plasticity in humans (Zatorre et al., 2007; Lappe et al., 2008; Herholz and Zatorre, 2012).

### Fractal Stimuli and Physiological Functions

One should also pay attention to the facts, which may indicate the influence of non-linear fractal factors on the fractal gait dynamics. Fractal dynamics of physiological processes, as described above, is an essential feature of a healthy organism. Destruction of fractal dynamics, including the rhythm of the gait, characterizes aging and some neurodegenerative disorders. We suggested earlier that dynamic fractal flickering may be a useful tool in the search for non-linear dynamics involvement in the activity of a visual system. And it may be a possible basis for new diagnostics and treatment of neurodegenerative diseases of the retina and brain (Zueva, 2013).

The fractal patterns of gait can be changed by synchronizing the gait dynamics with the fractal temporal structure of stimuli. Several studies have showed this ability for auditory (Hove et al., 2012; Kaipust et al., 2013; Uchitomi et al., 2013; Marmelat et al., 2014) and visual stimuli (Rhea et al., 2014a,b). Recent works (Rhea et al., 2014a,b) revealed that fractal patterns in the step-to-step intervals significantly changed during walking on a fractal visual metronome (flashing red square). However, the participants were not able to adequately reproduce the persistent fractal pattern that the stimuli exhibited. The experiment consisted of three phases: walking without the fractal stimulus, walking during entraining to a fractal visual stimulus, and walking with no stimulus. The fractal gait patterns of healthy young adults became enhanced during the synchronization phase. The effect remained after entrainment in the post-synchronization phase. The discrete fractal stimulus was noted to affect the retention better, causing a more persistent gait pattern in synchronization phase as compared to continuous fractal stimulus.

The results obtained in patients with PD can be considered as an evidence of the role that the temporal structure of the cue is essential to the sign or power of their effects on the CNS. Walking with fixed-tempo Rhythmic Auditory Stimulation can improve many aspects of the gait timing in PD patients (reviewed by Thaut and Abiru, 2010; see also Hove et al., 2012). However, this stimulation has been found to reduce rather than to increase the fractal scaling of step-to-step intervals (Hausdorff et al., 1997). Moreover, the stride variability becomes synchronized around a single frequency (Delignieres and Torre, 2009). Conversely, Hove et al. (2012) showed that the dynamic characteristics of the stride interval fluctuation in patients with PD can be improved to a healthy 1/f fluctuation level using interactive rhythmic cues. These authors emphasized that patients and healthy participants rarely synchronized the dynamics of gait with a fixed tempo Rhythmic Auditory Stimulation. When the synchronization occurs, the fractal scaling of gait patterns decreased far from healthy 1/f levels.

Uchitomi et al. (2013) recently studied the PD patients that were tested in four experimental situations: with the interactive rhythmic cue, fixed tempo-cue, 1/f fluctuating tempo-cue, and no cues. There was a significant effect of interactive rhythmic cues – the gait fluctuations of the patients gradually returned to a healthy level reinstating 1/f fluctuation while this did not happen in other circumstances. In the condition of interactive WalkMate, the cue rhythm was changed in response to the subject’s gait rhythms, that is, there was a mutual synchronization of the gait rhythms and cue rhythms via mutual entrainment. The authors suggest that mutual entrainment can facilitate gait relearning and expect a wider application of interactive rhythmic cues in the fields of rehabilitation.

In a review, 14 studies investigating whether a rhythmic auditory (music) cueing improves walking in patients with other neurological conditions than PD were analyzed (Wittwer et al., 2012). Moderate evidence of improving velocity and stride length in people with stroke due to gait training with rhythmic music were noted. Insufficient evidence for benefits of gait training using synchronization of walking to rhythmic auditory cues was found in HD, spinal cord injury, traumatic brain injury, dementia, multiple sclerosis, and normal pressure hydrocephalus. However, the authors suggest that the failure may be due to the poor methodological quality of some works (Wittwer et al., 2012).

Sejdić et al. (2012) studied the effects of different rhythmic sensory cues (aural, visual, and tactile) on the temporal dynamics of the healthy adult’s gait. These authors showed the greatest auditory rhythmic signal impact on walking parameters. However, the visual cue had no statistically significant effect on the scaling exponent.

Hunt et al. (2014) conducted a special study to find out whether the temporal structure of the complex auditory cue has different effects on the temporal pattern of the target behavior. The authors showed the ability to control the auditory–motor coupling by sound signals of different colored noise, which shift the temporal structure of the fractal gait dynamics to the statistical properties of specific signals.

Music perception is a complex cognitive task that involves the integration of the various structural components of music (melody, harmony, rhythm, tempo, and others). Different neural correlates have been associated with the music perception (Platel et al., 1997; Schmithorst et al., 2005; Gomez and Danuser, 2007; Lin et al., 2014). Hadjidimitriou et al. (2010) studied the EEG signals including the Mu rhythm in groups of advanced music students and non-musicians on the movements during the sound and audiovisual stimulation. Music students showed a significantly greater sensorimotor response at the auditory stimulation compared to non-musicians. At the audiovisual stimulation, the results were similar in both groups.

It is essential to note that these findings, on the one hand, may indicate a predominant role of professionally adequate stimuli (auditory) for musicians playing on the instruments. And, on the other hand, they do not exclude a smaller role of audiovisual integration in the modulation of locomotor activity in healthy individuals. Further investigations may determine the impact of audiovisual and auditory stimulation in aging and pathological conditions involving a reduction in fractal scaling of gait patterns in musicians and non-musicians.

## The Logical Substantiation of the Theory

### Key Facts and Regularities Derived from the Analyzed Studies include the following Highlights

• Nature is full of non-linear fractals that surround us throughout our lives.
• Humans evolved in a non-linear world, gaining experience in a complex multisensory environment.
• Fluctuations of physiological rhythms of a healthy body have the fractal properties and can be described using the theory of deterministic chaos.
• The fractal regulation of physiological processes is impaired with age and in diseases. Aging and pathological conditions tend to cause a loss of the complexity of many physiological processes, and quite often – to strict control of their fluctuation.
• The reduction in the multiscale complexity of the brain’s structure and activity characterizes the age-related neurodegenerative disorders of the brain and retina.
• Neuroplasticity plays a significant role in development, learning, memory, and in recovery from brain injury. Developmental plasticity includes changes in neural connections due to brain/environment interactions, and cellular changes induced by learning.
• The sensory deprivation that reduces or completely destroys the quality or quantity of a mono- and multisensory experience in the early brain development can alter the neural networks and functional connections that the brain continues to use in adulthood.
• At all levels of the CNS, adaptive or maladaptive plasticity may be caused by the loss or excess of mono- and multimodal stimulation and injury. It occurs as the consequence of a non-use or over-use or learning new skills.
• The brain plasticity decreases throughout the life, but the adult neuroplasticity can be reactivated in a variety of ways.
• The physical activity and exercise, cognitive training, increase in social interactions and visual stimulation, and other training programs related to the EE paradigm can re-activate the brain plasticity playing a crucial role in the improvement of cognitive function.
• Exposure to the auditory background (white) noise improves cognitive performance in inattentive people while it distorts the performance of attentive persons.
• The therapeutic approaches based on the SR improve the gait disturbance and cognitive performance in the patients with neurodegenerative disorders. Application of these approaches seems to be useful in the elderly and in the period of rehabilitation after a stroke.
• Architecture, painting, and musical compositions may have a fractal dimension.
• Listening to music and musical training have a positive impact on cognitive and motor functions, the brain activity, mood, and intelligence.
• Fractal patterns of gait can be changed by synchronizing the gait dynamics with the fractal temporal structure of sensory cues. The more prominent effect was proven for interactive rhythmic cues, suggesting that mutual entrainment can facilitate the gait relearning effectively.
• The temporal structure of the complex auditory signal has different effects on the temporal pattern of the target behavior. Auditory–motor coupling can be controlled by sound signals of different color types of noise, which transfer the temporal pattern of the fractal gait dynamics to the statistical properties of specific signals.

**Table 1** presents the summary of the current data, which are significant to the logical substantiation of the theory, and appropriate references.

**Table 1:** Current knowledge, which are significant to the logical substantiation of the theory.

Key facts and regularities	Reference
Nature is full of non-linear fractals that surround us throughout our lives	Feder (1988), Crownover (1995), Iannaccone and Khokha (1996), West et al. (1999)
Fluctuations of physiological rhythms of a healthy body have the fractal properties	Babloyantz (1989), Goldberger et al. (1990, 2002), Peng et al. (1993, 2002), Yamamoto and Hughson (1994), Hausdorff et al. (1995, 1996), Rabinovich and Abarbanel (1998), So et al. (1998), Faure and Korn (2001), Lipsitz (2004), Goldberger (2006), Izhikevich (2007), Mattei (2014)
The fractal regulation of physiological processes is impaired with age and in diseases with a loss of the complexity and often strict regularity of their fluctuation	Lipsitz and Goldberger (1992), Daxer (1993, 1995), Goldberger (1997), Hausdorff et al. (1997), Dingwell and Cusumano (2000), Avakian et al. (2002), Goldberger et al. (2002), Peng et al. (2002), Masters (2004), Abásolo et al. (2005), Bilney et al. (2005), Meigal et al. (2013), Nemati et al. (2013)
The reduction in the multiscale complexity of the brain’s structure and activity characterizes the injury and neurodegenerative disorders of the brain and retina	Saermark et al. (1989), Besthorn et al. (1995), Stam et al. (1995, 1996), Anninos et al. (2000), Kotini and Anninos (2002), Moolman et al. (2004), Abásolo et al. (2005), Breakspear (2006), Hornero et al. (2009), Hu et al. (2009, 2013), Dauwels et al. (2010), Takahashi et al. (2010), Ly et al. (2011)
Neuroplasticity plays an important role in development, learning, memory, and in recovery from brain injury	Abbott and Nelson (2000), He et al. (2006), Moucha and Kilgard (2006), Wierenga et al. (2006), Butz et al. (2009), Pascual-Leone et al. (2011), Wainwright and Galea (2013)
The reduction of mono- and multisensory experience in the early brain development alters the neural networks and functional connections that the brain continues using in adulthood	Hubel and Wiesel (1970), Gordon and Stryker (1996), Black (1998), Wallace et al. (2004), Wallace and Stein (2007), Feller (2009), Merabet and Pascual-Leone (2010), Putzar et al. (2010), Royal et al. (2010), Yu et al. (2010), Bengoetxea et al. (2012), Espinosa and Stryker (2012), Mereditha et al. (2012), Xu et al. (2012), Nagakura et al. (2013), Stein et al. (2014)
At all levels of the CNS, adaptive, or maladaptive plasticity may be caused by the loss or excess of mono- and multimodal stimulation and injury as the consequence of non-use or over-use and aging	Rapp and Heindel (1994), Perry (2002), Rhodes (2004), Burke and Barnes (2006), Mahncke et al. (2006), Peiffer et al. (2007), Diederich et al. (2008), Grady (2008), Stein and Stanford (2008), Holmes (2009), Yu et al. (2010), Mahoney et al. (2011), Maya-Vetencourt and Origlia (2012), Mozolic et al. (2012), Shors et al. (2012), DeLoss et al. (2013), Freiherr et al. (2013), Ramscar et al. (2014)
Disturbance of neuroplasticity plays a central role in neurodegenerative brain disorders	Lewis and González-Burgos (2008), Weber and Harman (2008), Hu et al. (2009), Porciatti and Ventura (2012), Varea et al. (2012), Wainwright and Galea (2013), Zhuang et al. (2013)
The brain plasticity decreases throughout life, but it was also found in adulthood	Dragoi et al. (2001), Chen et al. (2011), Rosa et al. (2013), Sur et al. (2013)
Adult neuroplasticity can be reactivated in aging and disease in a variety of ways The physical activity, cognitive training, increasing social interaction and visual stimulation, and other training programs can reactivate brain’s plasticity playing a crucial role in the improvement of cognitive function	van Praag et al. (2000), van Praag et al. (2005), Shors et al. (2001), Shors et al. (2012), Kempermann et al. (2002), Mahncke et al. (2006), Nithianantharajah and Hannan (2006, 2009), Mora et al. (2007), Maya-Vetencourt et al. (2008, 2011), Butz et al. (2009), Eysel (2009), Fisher et al. (2009), Levi and Li (2009), Smith et al. (2009), Baroncelli et al. (2010, 2012), Berry et al. (2010), Astle et al. (2011), Foster et al. (2011), Li et al. (2011), Spolidoro et al. (2011), Green and Bavelier (2012), Sale et al. (2012), Chapman et al. (2013), Mainardi et al. (2013), Merzenich (2013), Alwis and Rajan (2014), Prakash et al. (2014)
Exposure to auditory background noise improves cognitive performance in inattentive persons while it distorts the performance of attentive people	Sikström and Söderlund (2007), Söderlund et al. (2007), Söderlund et al. (2010), Anderson (2013)
The therapeutic approaches based on the stochastic resonance improve the gait disturbance and cognitive performance in the elderly, patients with neurodegenerative disorders, and in the period of rehabilitation after a stroke	Priplata et al. (2003), Yamamoto et al. (2005), Costa et al. (2007), Ross (2007), Pan et al. (2008), Ward et al. (2010)
Architecture, painting, and musical compositions may have a fractal dimension	Hsü and Hsü (1990, 1991), Briggs (1992), Bovil (1996), Hazard et al. (1998–1999), Oswald (2001), Taylor et al. (2002), Batty (2005), Barnett (2009), Forsythe et al. (2011), Harris (2012), Lu et al. (2012)
Listening to music and musical training have a positive impact on cognitive and motor functions, the brain activity, mood, and intelligence	Petsche (1996), Campbell (1997), Yuan et al. (2000), Jenkins (2001), Bhattacharya and Petsche (2005), Jausovec et al. (2006), Bugos et al. (2007), Särkämö et al. (2008, 2014), Fink et al. (2009), Kowatari et al. (2009), Parbery-Clark et al. (2009, 2011), Berkowitz and Ansari (2010), Kraus and Chandrasekaran (2010), Sink et al. (2011), Ellamil et al. (2012), Herholz and Zatorre (2012), Zendel and Alain (2012), Anderson (2013), Alain et al. (2014), Benoit et al. (2014), Fink and Benedek (2014)
Fractal patterns of walking can be changed by synchronizing the gait dynamics with the fractal temporal structure of sensory cues	Hove et al. (2012), Sejdić et al. (2012), Wittwer et al. (2012), Kaipust et al. (2013), Uchitomi et al. (2013), Marmelat et al. (2014), Rhea et al. (2014a,b)
The temporal structure of the complex auditory signal has different effects on the temporal pattern of the target behavior. Auditory-motor coupling can be controlled by sound signals of various types of noise, which shift the temporal pattern of the fractal gait dynamics to the statistical properties of signals	Sejdić et al. (2012), Hunt et al. (2014)

### Logical Conclusions that We Draw from a Comparison of Scientific Observations Gained in Various Fields of Research

The experience of human evolution in a non-linear world and the interactions of humans throughout their life with a non-linear environment permit us to assume that these factors underlie the brain plasticity resulting from this experience. These factors can underlie the management of external inputs through different modalities. We believe that not only the quality and quantity of sensory information, but its non-linear fractal structure in many spatial and temporal scales is important for the health of the brain and is involved in fractal regulation of biological rhythms.

Different studies have shown the principal ability to reactivate adult neuroplasticity in a healthy aging and age-dependent CNS pathologies in a variety of ways through learning experiences and physical exercise. So, it is logical to assume that this experience can be of greater benefit if it involves the interaction of sensory brain structures with mono- or multimodal stimuli having time-invariant fluctuations of their parameters. The fractal patterns of gait were shown to change by synchronizing the dynamics of gait with the fractal temporal structure of auditory rhythm and with interactive rhythmic cues. We assume that the fractal audio-visual and sensory-motor stimulation may involve other mechanisms of fractal regulation of body rhythms compared to the direct coupling (synchronization) of the rhythms discussed in the recent articles. More probable is that fractal audio-visual stimulation presents an effective way to improve sensory processing, cognitive and motor function through the reactivation of brain plasticity.

Art can heal, changing the human physiology and perceptions of the world. Nevertheless, the results are not the same in different studies. The short-term effects (or the absence of effects) of Mozart’s music on the performance and brain physiology may be linked to the fact that all the researchers used the passive listening to Mozart’s music. In other studies, the effects of musical training during the solution of perceptual and motor tasks were associated with structural and functional changes that occurred mostly in the brain of musicians when compared to non-musicians.

It is essential to note that these findings, on the one hand, may indicate a predominant role of professionally adequate stimuli (auditory) for musicians playing on the instruments. And, on the other hand, they do not exclude the smaller role of audiovisual integration in the modulation of locomotor activity in healthy individuals. Further investigations can probably determine the effect of audio-visual and auditory stimulation in an aging musicians and non-musicians, as well as in pathological conditions associated with a reduction in the fractal scaling of the gait rhythm.

Various studies also encourage us to pay attention to the fact that the positive effect of music on the perceptual learning and higher cognitive functions depends on their initial distortion. The effect is better for the elderly than young adults and patients with neurodegenerative disorders than in normal aging. The healing effect of music on the auditory, visual and motor processing, cognitive and emotional state seems to be more prominent in different pathologies of the CNS than in normally aging individuals.

Similar to the results of musical training, we should expect the less pronounced effects of different cognitive and perceptual training in young healthy compared to the elderly or individuals suffering from age-related diseases. The capacity of homeostatic mechanisms in a healthy subject should be apparently sufficient to withstand the environmental perturbations of moderate strength. Otherwise, the detection of a significant shift in the characteristics of sensory and motor functions in young healthy individuals perhaps would be more correctly considered as an evidence of potentially damaging rather than therapeutic effect.

We can also presume that fine arts and music created by great masters may have fractal properties and potential curative impact on the person who actively perceives these works by passing them through his heart and brain. On the other hand, active participation in creative activity can alter the brain functioning, and, as a result, change the life. The results that showed the effects of music on creative thinking and emotional state, which mediate our perception of the world, may evidence this assumption.

Background noise stimulation increases arousal and performance of inattentive people but reduces the performance of attentive persons. One cannot exclude that the sign and power of the background music impact on cognitive function may also depend on individual characteristics, in particular, the strength of the internal neural noise.

### Factors that may Reduce the Complexity of the World Picture Painted by the Brain

The human brain needs to obtain the complex multi-sensory experience during a lifetime. The distortion or diminishing of our sensory experience may lead to a disruption of the perfect complex structure, connectivity and functioning of the brain in the early development, or may underlie neurodegenerative disorders in the old age. The inherent complexity of the human sensory perceptions and the integration of multimodal sensory information from the individual channels in a holistic perception of the world are well-proved now. It allows to suggest that for the healthy brain, maintenance and preservation of the complexity and rich diversity of environmental stimuli that accompanies humans from birth and throughout the life are also critically important.

In the surrounding world, the linear stimuli with the ordered temporal or spatial structure are not usually effectively affecting people because they are not typical for our natural habitat. However, there are situations in which we are subjected to a more or less prolonged exposure to monotonically structured artificial environment. Formation of these conditions might be related to private life conditions of a person. For example, in a risk category one can include the residents of cities with a limited diversity of the visible landscape, people with the reduced mobility and possibility to change scene – the disabled, chronically ill, institutionalized patients. In a separate group, one should include elderly subjects suffering from not only gait disturbance, but also having visual and hearing impairment. In the old age, there is a sharp narrowing of the diversity of experiences and a decrease in the total flow of sensory information available to man. It limits the perception of the complexity of the world around them.

Deficiency or loss of complexity of sensations and images created by the brain may occur under the following conditions:

(1) A decline in the intensity of light reaching the retina reduces or distorts the perception of geometric and dynamic fractals of nature.
(2) A decrease in image contrast alters not only the perception of the details of the observed object, but also its geometric complexity. In amblyopia associated with the reduction of subjective brightness and contrast of the image, one can expect the positive impact of the therapeutic fractal stimulation.
(3) In the cases of transparent optical media and standard eye refraction, the reduction of complexity of the images processed by the retina and brain, can apparently occur:

• In the pathology of the retinal ganglion cells (glaucoma, optic neuritis of different etiology, etc.), when distorted information is sent by the retinal ganglion cells to the lateral geniculate nucleus and visual cortex;
• In pathological conditions, which alter the level of internal noise of the retina;
• In neurodegenerative diseases, such as glaucoma and AD, when the simplification of visual processing likely reflects the simplification of neural networks and loss of complexity in the functional activity of the brain and retina.

(4) The monotony of visual and other sensory features of the habitat:

• Low levels of life and culture: deficit of impressions and emotional experiences;
• Urbanization problems: deficit of diversity in architecture;
• Low levels of social communication: deficit of the diversity of experience;
• A monotonous work: deficit of creativity.

In all these situations, the diversity of living conditions can play an important role. The effect is expected to be greater when involving the multisensory integration.

### Possible Applications and Future Research

**FIGURE 1:**
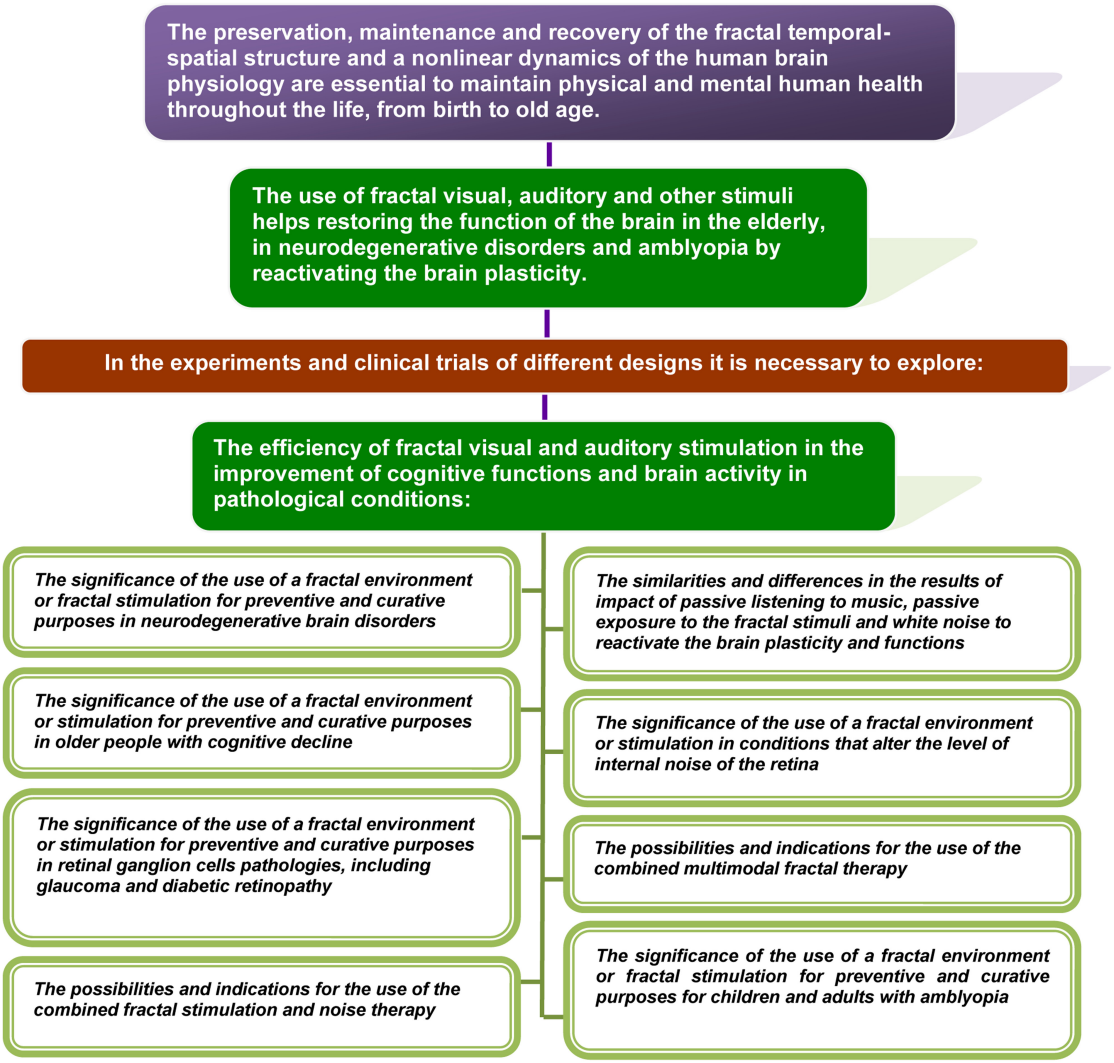
**The directions for future studies based on the theory of ‘Fractality of sensations’ to explore benefits of non-linear stimulation and a scope of its application**.

**FIGURE 2:**
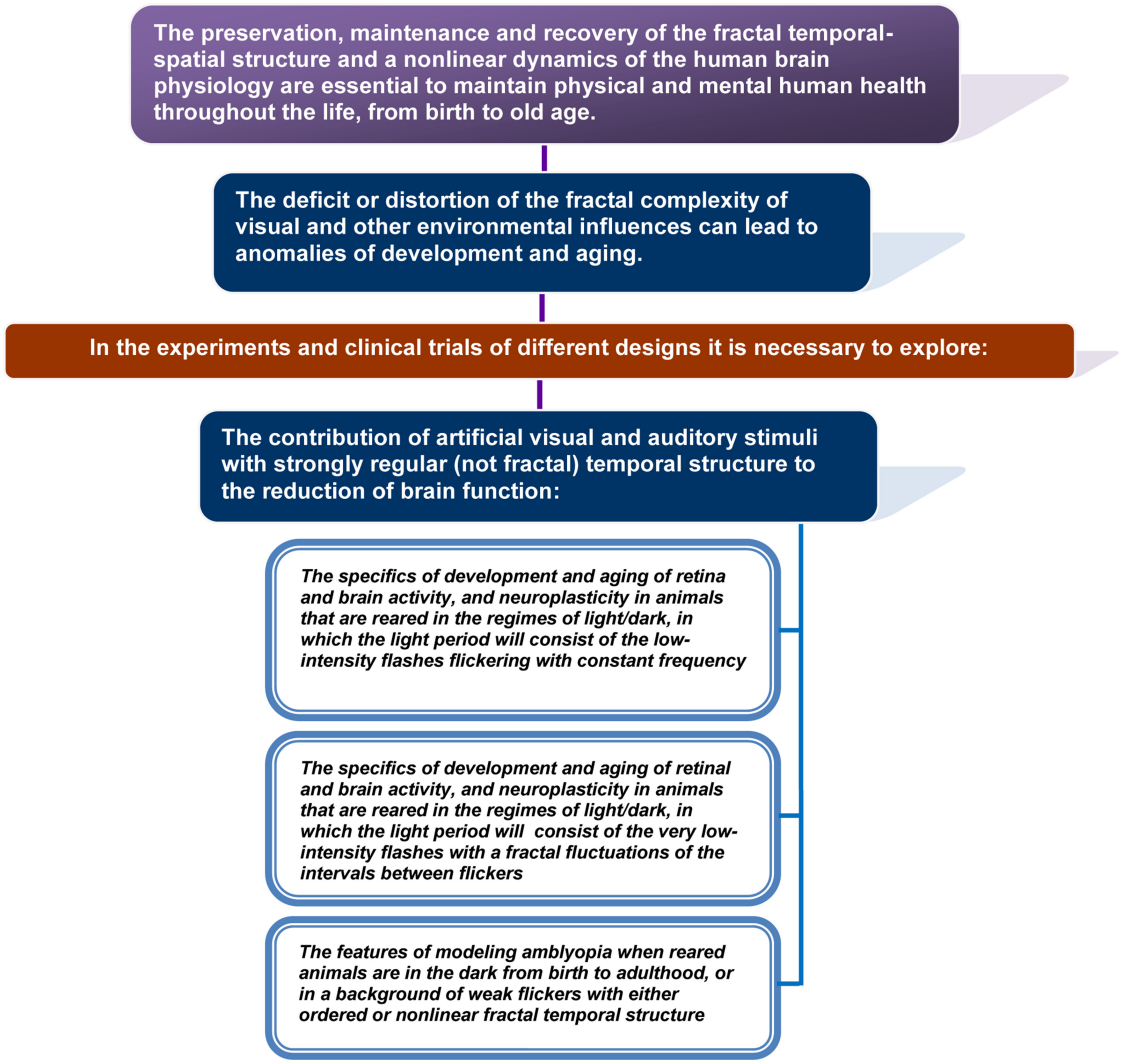
**Possible directions for future studies based on the theory of ‘Fractality of sensations’ to explore adverse effects of deficit in fractal stimulation**.

The presented theory provides the basis to open new directions for scientific studies. These studies primarily should be designed to test the usefulness and validity of the assumptions that are closely related to and follow directly from the declared theoretical relationships. **Figures 1** and **2** indicate some directions for future studies based on the theory of ‘Fractality of sensations’ to explore benefits of non-linear stimulation and a scope of its application and adverse effects of deficit in fractal stimulation.

The efficiency of fractal visual and auditory stimulation in the improvement of cognitive functions and brain activity in pathological conditions is necessary to be estimated in the experiments and clinical trials of different designs (**Figure 1**).

One should compare the impact of mono and multisensory fractal therapy and the known effect of white noise described in the elderly, patients with PD and inattentive persons. It is desirable also to explore the possibilities of and indications for the combined use of fractal stimulation and noise therapy.

For children with amblyopia and older people with cognitive decline, it is especially important to ascertain the significance of the use of a fractal environment or fractal stimulation for preventive and curative purposes.

One should also explore another assumption related to the theory that exposure to artificial visual and auditory stimuli with strongly regular (not fractal) temporal structure may contribute to the reduction of brain function (**Figure 2**). The mechanisms of revealed adverse effects would be necessary to describe and explain.

It seems important to assess consequences of urbanization and positive impact of the new art in the architecture of the cities and their lighting to provide non-linearity of ambient human artificial environment closer to the natural conditions. The habitat change changes a man.

Future experimental and applied research in these and related areas could provide opportunities to confirm or deny the validity of the predictions contained in the theory.

### Weaknesses in the Theory to Clarify in Future Studies

It is unlikely that all the wealth of sensations that a natural environment gives us is limited only to structures with the properties of temporal and spatial fractals. It is impossible to equate the world in all its diversity with a deterministic chaos in the nature. The spatiotemporal structure of various natural stimuli is diverse and may include low-dimensional rhythms, differing degrees of randomness. All the factors that accompany man in his evolution and the early periods of brain development have to play a role in the formation and self-organization of the CNS, and in the mechanisms of neuroplasticity. There is a marked difference between the passive human exposure to natural stimuli during the life and active perception of spatial–temporal stimuli. In the latter case, one should underline the significance of a dynamic behavior and creative thinking.

Existing knowledge does not yet allow us to assume how different the impact of the artificial fractal rhythms in comparison to white noise and cognitive and physical training on patients with amblyopia and neurodegenerative diseases can be. It seems most likely that the greatest potential benefit can be expected from the combination of these factors, which may vary for different pathological conditions, and this has to be substantiated and proved experimentally. One should explore how making a passive training effective for those who, for example, cannot play a musical instrument or actively enjoy music. The same problem may exist with the usage of artificial fractal stimulation that is always passive. It is interesting to investigate the similarity in the effects of passive listening to music and passive exposure to the fractal stimuli, and the impact of white noise.

Future studies should also answer the following distinct questions. What kinds of mono or combined stimulation will help to overcome the adverse effects of the increased level of internal noise in the sensory system and the brain of patients and the elderly? What are the mechanisms and laws of interaction of intrinsic neural noise and external noise? Clarification of these issues is expected to define or limit the range of conditions that best meets the rules formulated in the theory of “fractality of sensations.”

## Conclusion

We proposed for the first time in this article that temporal and spatial structures of visual and other sensory signals, which affect us throughout the life, are crucial for the normal development and aging of the brain. Conversely, the absence of fractal sensations is supposed to promote distortion of brain functioning and reduce the capacity of the adaptive plasticity.

Known facts related to different aspects of the problem have been considered. Described literature data and logical analysis allowed theoretical substantiating and formulation of a theory named “fractality of sensations.” The theory establishes relationships between the normal functioning and pathology of the brain and visual system, and the spatial–temporal structure of the visual and other sensory stimuli that affect people throughout their life. The theory argues that the deficit or distortion of the fractal complexity of visual and other environmental influences may lead to anomalies of development and aging. Application of fractal flickering and other fractal rhythms helps to restore the function of the brain and visual system, particularly in the elderly, in patients with neurodegenerative disorders, as well as with amblyopia.

We outlined here possible applications, experimental and applied research, based on the key tenets of the theory, as well as the weaknesses of the theory, which need to be clarified in future studies. Since the average life expectancy increases, the prevalence of age-related neurodegenerative diseases is also steadily increasing. Thus, the creation of new non-pharmacological methods of therapy that may slow cognitive decline, as well as weaken the manifestation of neurodegenerative diseases is an imperative. Development of new practical training paradigms based on the principles outlined in the theory may be useful in the prevention of cognitive decline, treatment, and rehabilitation of various pathologies of the brain. Also, the fundamental tenets of the theory can be used to mitigate the social problems associated with the objective limitations of the fractal complexity of sensations in particular categories of people.

## Conflict of Interest Statement

The author declares that the research was conducted in the absence of any commercial or financial relationships that could be construed as a potential conflict of interest.

## Abbreviations

AD: Alzheimer’s disease
DR: diabetic retinopathy
EE: environmental enrichment
EEG: electroencephalography
HD: Huntington’s disease
PD: Parkinson’s disease
PERG: pattern electroretinogram
SCN: suprachiasmatic nucleus
SR: stochastic resonance
